# The process and perspective of serious incident investigations in adult community mental health services: integrative review and synthesis

**DOI:** 10.1192/bjb.2023.98

**Published:** 2025-02

**Authors:** Helen Haylor, Tony Sparkes, Gerry Armitage, Melanie Dawson-Jones, Keith Double, Lisa Edwards

**Affiliations:** 1 First Response Crisis Service, Bradford District Care NHS Foundation Trust, UK; 2 Faculty of Management, Law and Social Sciences, University of Bradford, UK; 3 Research and Development Department, Bradford District Care NHS Foundation Trust, UK; 4 Faculty of Health Studies, University of Bradford, UK; 5 Library and Health Promotion Resources Centre, Bradford District Care NHS Foundation Trust, UK; 6 Patient and Carer Experience and Involvement Team, Bradford District Care NHS Foundation Trust, UK

**Keywords:** Suicide, community, serious incident investigation, organisational learning, patient context

## Abstract

**Aims and method:**

Serious incident management and organisational learning are international patient safety priorities. Little is known about the quality of suicide investigations and, in turn, the potential for organisational learning. Suicide risk assessment is acknowledged as a complex phenomenon, particularly in the context of adult community mental health services. Root cause analysis (RCA) is the dominant investigative approach, although the evidence base underpinning RCA is contested, with little attention paid to the patient in context and their cumulative risk over time.

**Results:**

Recent literature proposes a Safety-II approach in response to the limitations of RCA. The importance of applying these approaches within a mental healthcare system that advocates a zero suicide framework, grounded in a restorative just culture, is highlighted.

**Clinical implications:**

Although integrative reviews and syntheses have clear methodological limitations, this approach facilitates the management of a disparate body of work to advance a critical understanding of patient safety in adult community mental healthcare.

The quality of serious incident investigations and subsequent organisational learning are international patient safety priorities, yet improvement work appears slow.^1^ Any suicide in England that occurs when the deceased was part of an open episode of mental healthcare is a reportable incident.^2^ In 2021, 5583 suicides were registered in England and Wales.^3^ The impact of the COVID-19 pandemic on suicide rates is complex, and any hypothesised increases, particularly early in the pandemic, have largely been unfounded through national and international research.^4–7^ However. ongoing analysis is crucial,^7^ particularly in regard to the consequences of a ‘coming global economic downturn’.^8^

There is growing empirical literature detailing the severe, longstanding effects of bereavement by suicide,^9^ and a call for more robust evidence-based interventions.^10^ It is also apparent that mental health clinicians are negatively affected,^11^ including those involved in the investigation process.^12^ Internationally, despite some reduction in suicide rates since 1990, these deaths continue to be a significant contributory factor in global mortality.^13^ A zero suicide framework (ZSF) is gaining popularity worldwide.^14^ This inclusive approach demands that all involved in service provision regard each suicide as preventable. The seven elements of the ZSF are summarised in Box 1.

Box 1 Summary of the seven elements of the Zero Suicide Framework (ZSF)^14^

Lead: **System change occurs with sustained and committed leaders who learn and improve practices following adverse events.**

Train: **Train all staff—clinical and non-clinical—to identify individuals at risk and respond effectively, commensurate with their roles.**

Identify: **Screen and assess every new and existing patient for suicidal thoughts and behaviours in an ongoing and systematic way using standardized tools.**

Engage: **Patients at risk for suicide agree to actively engage in a package of evidence-based practices that directly targets their suicidal thoughts and behaviours.**

Treat: **Utilize evidence-based treatments that focus explicitly on reducing suicide risk to keep patients safe and help them thrive.**

Transition: **Put policies into action that ensure safe hand-offs between caregivers and upon discharge.**

Improve: **Apply data-driven quality improvement. Use data to inform system changes that will lead to improved patient outcomes and better care for those at risk.**

Although suicide prevention is a National Health Service (NHS) priority in England,^15^ the UK National Confidential Inquiry into Suicide and Safety in Mental Health (NCISH) found 27% (*n* = 18 403) of the general population who took their own lives in England during 2010–2020 had mental health services contact within 12 months.^5^ Just under half (46%) had contact 7 days before death.^5^

The increased risk of suicide post-discharge from in-patient psychiatric care is well documented in the literature;^16^ Chung et al's meta-analysis^17^ describes the risk as ‘extraordinary’ and requiring greater attention upon ‘safe transition from hospital to community care’.

Although there is evidence of a decline in suicides that occur within psychiatric in-patient settings,^5^ the following intersecting factors mean that it is imperative that adult community mental health services are made a priority for suicide prevention research. There has been a reduction of around 50% in in-patient bed numbers over the previous two decades,^18^ contributing to increased pressure on community-based services. Also, in the UK, the various structural changes following reform of mental healthcare provision has added to the multiple service transitions that people in distress and their carers are required to negotiate.^19–21^ Of further concern, a significant proportion of those people in distress will have complex needs,^22^ spending considerable time away from the direct observation of professionals. Transitions of care are well recognised as key patient safety issues in physical healthcare research,^23^ but are only a recent focus within mental healthcare research.^24^ The British Government's white paper,^25^ which aims to redress compartmentalised service delivery, evidences the more challenging context of contemporary community care.

Development of patient safety driven learning systems are national and international concerns.^26,27^ Over a decade ago, UK policy makers first instituted a National Framework for Reporting and Learning from Serious Incidents Requiring Investigation,^28^ which was later underwritten by the Serious Incident Framework (SIF).^29^ The SIF has since attracted criticism in its ability to deliver high-quality investigations, communications and learning;^30^ particularly with regard to the centrality of root cause analysis (RCA), which was defined as: ‘a systematic process whereby the factors that contributed to an incident are identified. As an investigation technique for patient safety incidents, it looks beyond the individuals concerned, and seeks to understand the underlying causes and environmental context in which an incident happened'.^29^

In response to these concerns, the NHS Patient Safety Strategy developed a new vision for improving patient safety by focusing on organisational culture and systems. Driven by the strategic aims of ‘insight’, ‘involvement’ and ‘improvement’,^31^ the Patient Safety Incident Response Framework (PSIRF)^32^ was launched as a replacement for the troubled SIF.

Informed by collaborations between NCISH and the Serious Incident Review Accreditation Network,^33,34^ the PSIRF^32^ marks an intended shift in the way that the NHS undertakes investigations, not least by a move away from RCA, but also a clear paradigmatic shift from Safety-I to Safety-II.^35^ This shift involves moving from a retrospective to a prospective approach; recognising the reality of routine practice; variability in human performance; and learning from human adaptation and success, rather than failure alone. RCA has not been replaced by a specific investigative method in the PSIRF, but by a range of methods suited to a systems approach.^36,37^

The above initiatives illustrate a drive to effectively engage patient safety approaches to aid suicide prevention, but they have not yet been exposed to robust critical appraisal. We argue that there is no national or international evidence base for investigating suicides. A pragmatic narrative review focused on suicide investigations, recommending ‘Six Steps’ to improve learning, was recently published.^38^ However, the review did not differentiate between in-patient and community suicides or empirical and non-empirical work, and did not involve a formal synthesis. In a recent narrative review, Averill et al^39^ document their concerns about the lack of research about patient safety in the context of community mental health services. They call for the need to better understand the patient journey and what counts as a preventable safety issue, and the need to challenge current risk management approaches that situate risk within the person. We believe there is a strong need to systematically examine the literature on the investigative process following suicide in adult community mental healthcare.

In view of the literature's inherent uncertainties and inconsistencies, an integrative review and synthesis was undertaken. The aim was to critically explore investigative approaches with the following objectives: (a) to determine the nature and extent of relevant individuals’ involvement in the investigative process; (b) to appraise the strengths, limitations and evidence base underpinning the approaches taken; and (c) to consider the influence of various investigative approaches on organisational learning.

## Method

Because of the paucity of empirical literature in this area, an integrative review^40^ of empirical and non-empirical peer-reviewed literature was undertaken, to generate a broader and more nuanced understanding of the complexities inherent in this domain. Integrative reviews are indicated where the knowledge base is relatively modest, and where new perspectives can help advance what is known.^41,42^ The review incorporates a narrative synthesis, undertaken in an iterative style following Popay et al,^43^ as a way of structuring and making sense of the text.

The review team comprised researchers (H.H., T.S., G.A.), carers (L.E., K.D.) and an information specialist (M.D.-J.). The search strategy targeted international, English language literature, using the National Service Framework for Mental Health (NSFMH)^21^ as a temporal backstop because of its commitment to suicide prevention. Title/abstract keyword searches of CINAHL, Medline, PsycINFO, Cochrane Database of Systematic Reviews and EMCare databases were undertaken. Keywords (Table 1) were derived from preliminary readings arising from the review protocol. To maintain fidelity with the objectives of the study, exclusion criteria were applied (Box 2).

**Table 1:** Search terms

Concept 1	AND	Concept 2	AND	Concept 3
exp SUICIDE/ suicid*.ti mental.ti psychiatry*.ti		‘adverse incident*’.ti ‘adverse event*’.ti ‘serious incident*’.ti ‘serious event*’.ti ‘serious untoward’.ti ‘sentinel event*’.ti ‘critical incident*’.ti ‘critical event*’.ti ‘system err*’.ti harm*.ti ‘avoidable death*’.ti ‘preventable death*’		cultur*.ti,ab leadership.ti,ab process*.ti,ab systems.ti,ab

Box 2 Exclusion criteria

**Table:** 

• Learning from practices that fall outside of internal service review/audit activities.
• Specific patient populations and/or specific clinical conditions.
• Specific professional groups.
• Self-harm.
• Hospital (non-community) based mental healthcare.
• Service-level cultural context of serious incident investigations and how they are reported.
• Outcomes, recommendations and implementation of recommendations in relation to suicide investigations.

H.H. and T.S. independently reviewed titles and abstracts before iteratively agreeing a final inclusion list. In case of disagreement, the full text was subject to detailed scrutiny, with G.A. adjudicating where needed. This resulted in ten articles, categorised as empirical (*n* = 5) or non-empirical (*n* = 5). Bibliographic cross-referencing highlighted a further six relevant articles, three empirical and three non-empirical. Cross-referencing offered an organic dimension to the search strategy,^44^ reaching an agreed saturation point.^45^ The process is captured in the table below (Table 2).

**Table 2:** Literature selection process (adapted from Page et al^59^)

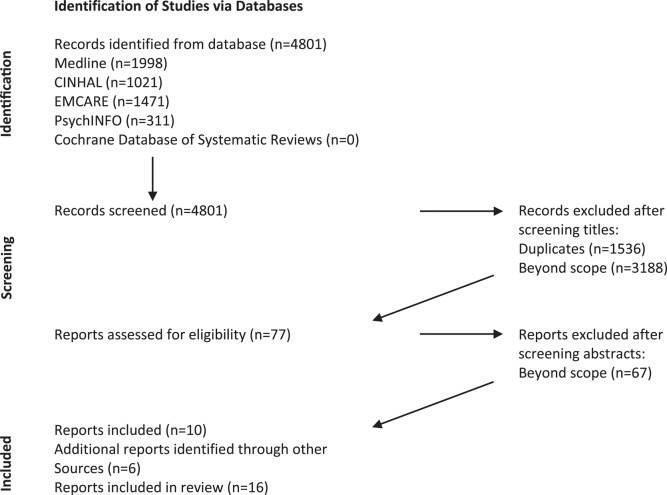

Wyder et al^46^ undertook a systematic narrative meta-synthesis, and although there is methodological debate as to whether such research is considered primary or secondary, it is included in the empirical section because of the novel insights afforded.^47^

### Model of synthesis and process

Following Popay et al,^43^ we adopt a particular model of synthesis, where there is a focus on text as a vehicle for making sense of the literature (see Box 3). Initially, H.H., T.S. and G.A. independently read the identified literature and recorded tentative patterns or themes.^48^ The reading was inductive, deductive and reflexive;^49^ adding interpretive value and authenticity to the synthesis.^50^ Reflecting the different approaches to empirical and non-empirical work, two separate summaries were centrally recorded to facilitate synthesis (see Supplementary Appendix 1 available at https://doi.org/10.1192/bjb.2023.98).

Box 3 Elements of synthesis (following Popay et al^43^)

Stage 1 Developing a theoretical model of how interventions, work, why and for whom

Stage 2 Developing a preliminary synthesis

Stage 3 Exploring relationships in the data

Stage 4 Assessing the robustness of the synthesis product

Throughout the analysis H.H., T.S. and G.A. discussed, revised and agreed thematic development regarding relevant individuals (who): the practice context (how) and the aim of the underpinning approach of the serious incident investigation (why). Relevant individuals included carers, the clinicians involved, the investigative team and the organisation itself. The practice context drew upon several intersecting themes, often influenced by policy, theory and practice developments. To generate learning for both the organisation and carers, investigative approaches, as with the prominence of some relevant individuals, appeared responsive to different influences (national policy, for example) over time (Box 4).

Box 4 Thematic patterns

1. Early development of approaches to serious incident investigations
2. Dominance of root cause analysis and related critiques
3. The context of complexity
4. Competing hierarchies of knowledge; technical–rational and experiential
5. Development of a patient safety agenda

We undertook a quality analysis of the empirical studies using the Mixed Methods Appraisal Tool (MMAT).^51^ We are unaware of a non-empirical quality assessment tool and consequently undertook our own appraisal. We also engaged in a critical reflection of our own review and synthesis processes.

### Results

Empirical literature is international and dominated by UK and Australian authors, most having been published after 2018. Empirical work stems from academic and healthcare sources, both singularly and collaboratively. The empirical papers are primarily qualitative and draw upon various methodologies and methods. In contrast, most non-empirical work emanates from the UK and largely precedes 2008.

#### Early development of approaches to serious incident investigations

Amos and Shaw,^52^ Catalan et al,^53^ Clarke,^54^ Neal et al^55^ and Rose^56,57^ provide a range of editorial, commentary and opinion/debate pieces, offering background commentary on the development of serious incident approaches to learning from deaths in the UK. The relocation of mental healthcare from hospital to community coincided with several high-profile media cases in the 1990s and a period of evidence-based modernisation, both in service delivery,^21,58^ and in addressing the quality of learning following adverse events (including suicide) reflecting emergent NHS policy at the time.^60,61^ Rose^56^ describes an early systematic process, charged with reviewing and learning from serious incidents. Two independent senior clinicians from outside the host trust facilitated case presentations for team members involved with the case. Groups were inclusive, interprofessional and designed to stimulate ‘peer group discussion’.^56^ The approach was an attempt to routinely prioritise, but also reduce the risk of serious incidents through a collaborative, micro-level analysis. Poor access to resources, process inertia, carer uncertainty and filing comprehensive reports for each case were seen as disadvantages. Rose^56^ indicates that although the process involves external facilitators, reviews are essentially in-house and consequently there is a risk of bias. Nevertheless, the process does illustrate the importance of achieving a balance between generating high-quality learning when supporting staff.

Amos and Shaw's^52^ editorial acknowledge the strengths and limitations of Rose's^56^ work against national-level inquiries.^62^ They argue that local reviews can harness a more inclusive interprofessional understanding of the local context that national studies may neglect. In contrast, and in response to plans to build expertise in RCA within the NHS,^61^ Neal et al^55^ cite Rose^56^ as an exemplar of the utility of independent investigation (operating regionally or nationally) to develop expertise. Catalan et al^53^ describe an alternative local, multidisciplinary audit, running alongside a management-led process. In a later article, Rose^57^ re-evaluates his previously reported review process in context of UK NHS developments, including the NSFMH.^21^ The rise of formal management procedures, the increasing complexity and diversity of service delivery, and the shifting emphasis to the analysis of systems rather than individuals are identified as three intersecting aspects of a new interest in patient safety.^58^

Although Rose^57^ discusses collaborative processes between management and front-line approaches, Cohen's^63^ editorial argues that this can be subsumed by other organisational priorities, and that the structure and organisation of management-led investigative processes defend against a range of anxieties that may undermine organisational objectivity. Turner et al's^64^ conceptual work echoes Cohen's^63^ earlier concerns by moving away from a management-led RCA approach, and advocate a more transparent, inclusive and ultimately healing process.

#### The dominance of RCA and related critiques

Over 80% of the literature locates RCA as the dominant approach. Summaries of RCA are provided by Vrklevski et al's^65^ empirical work and Clarke's^54^ conceptual analysis. RCA has been lauded for its ability to provide a systematic response to incidents,^65^ without it being perceived as threatening, instead focusing upon mutual learning, systems and processes.^55^ Yet, despite its dominance within the literature and long-standing application, concerns about the utility of RCA when investigating suicides are clear. Concerns have been present for some time,^54,55^ but not addressed.

The principal criticism of RCA concerns the oversimplification of causation.^54,64^ The phrase itself is misleading, not only carrying the presumption of a single cause giving rise to the incident, but one that also assumes a linear notion of causality.^55^ Moreover, Vrklevski et al^65^ indicate that within mental health services, root causes are rarely found and, based on their analysis of the evidence base, argue that it is ill-suited to investigating low-frequency complex events such as suicide. Gillies et al^66^ and Canham et al's^67^ empirical work argues that RCA follows an oversimplified understanding of the relationship between events, and fails to appreciate the complexity and unpredictability of human behaviour. As an example of the complexity that may be missed by RCA, Turner et al^64^ draw upon the work of Hollnagel et al^68^ and Funabashi et al^69^ to call for a better understanding of the gap between policy and front-line practice, termed as ‘work as imagined’ versus ‘work as done’. Additionally, several authors indicate a range of cognitive biases that can further impede the RCA process.^65^

More broadly, Fröding et al's^70^ study asserts that RCA is bounded by a micro-systems approach with a narrow focus upon the interaction between the patient and clinicians at final contact. This point is echoed by Turner et al,^64^ who suggest that it sidesteps and undermines the broader context of the patient journey.

Vrklevski et al^65^ assess the evidence base underpinning the effectiveness of RCAs, describing the empirical literature as both ‘sparse’ and ‘limited’, concluding that RCA is ‘not the model of best fit’. Fröding et al,^70^ Turner et al^64^ and Vrklevski et al^65^ argue that the process generates weak recommendations that are unlikely to affect practice. Fröding et al^70^ comment on the weak pull of recommendations relating to the actions of individual staff members, rather than systemic recommendations. In their empirical work, the authors also found that recommendations sometimes involve potentially unsustainable changes to existing practices, showing little effect on suicide rates despite significant resource investment. Turner et al^64^ propose that RCA findings may promulgate a risk-averse culture, which in turn may promote more restrictive practices and run counter to progressive innovations such as positive risk-taking.^71^ In contrast, some sections of the literature did not raise concerns about the RCA approach, proposing that it can provide valuable insights.^46,72^ In a somewhat contradictory analysis, Odejimi et al's^72^ research claims that RCA cannot offer ‘conclusive evidence’ of the factors contributing to suicide, but can ‘provide an indication of the underlying causes of suicide’.

#### A context of complexity

The notion of complexity as a defining characteristic of community services and, in turn, understanding suicide is clear in the literature. This theme has three broad strands: services themselves, suicide risk assessment and carer involvement in investigations.

The link between services is not only identified as a challenge for practitioners engaged in suicide prevention work,^46,57^ but is also a challenge for patients and their carers as they seek access to meaningful, responsive and timely care. In their systematic review of suicide deaths, Wyder et al^46^ report that patients often move between services when in crisis, posing multiple challenges for information sharing. They also note that services frequently shift in design and composition.

Beyond the hospital setting and in the community, suicide is identified by Fröding et al^70^ as being significantly different from other forms of harm, as it is a ‘final outcome of several interacting factors over time’, inevitably occurring away from the oversight of mental healthcare professionals.

Canham et al^67^ report that community services face significant challenges in the management of suicide risk. First is the issue of monitoring risk in the absence of constant observation. Second, unpredictable engagement with patients threatens the availability of clinical feedback. Third, clinical decision-making about new patients is based on limited knowledge. Fourth, treatment does not always fit patient preference. Finally, communication can be undermined when the care process involves several multidisciplinary teams, across multiple sites, at different times. Canham et al^67^ question the capacity of services to respond to crises and suggest that increasing capacity may be undermined by ‘lean thinking’ orthodoxies. Gillies et al,^66^ Vrklevski et al^65^ and Wyder et al^46^ corroborate Canham et al's^67^ observations regarding the availability of risk information to clinicians as partial and variable.

Jun et al^73^ interviewed clinicians about their approaches to suicide risk assessment, drawing attention to the knotty decision-making involved. Their findings suggest that tension exists in balancing clinical need with patient wants; personal, professional and organisational resources; legal and procedural responsibilities and constraints from legislative and regulatory influence.

Several papers also question the predictive ability of suicide risk assessment (Fröding et al,^70^ Gillies et al,^66^ Neal et al,^55^ Odejimi et al,^72^ Turner et al,^64^ Vrklevski et al^65^ and Wyder et al^46^) in such a complex clinical setting. Turner et al^64^ draw upon the dark irony that exists when investigations flag inadequate risk assessment as contributory, when evidence simultaneously indicates the fallacy of suicide risk prediction. Moreover, the authors argue that asserting demands for better risk assessment not only reinforces the myth, but undermines other potential areas of inquiry.

Although there was recognition that carer involvement facilitates transparency in the investigative process,^54^ Bouwman et al's^74^ later work provides empirical evidence for the role of carers as a potential source of information when attempting to make sense of the complexity associated with suicide investigation. From a Dutch perspective, the authors identified a lack of research and policy guidance concerning carer involvement, with the investigative process being professionally dominated. Limited carer involvement was countered by recognition that carers may not wish to participate, or feel unable to, particularly in the period close to their loss. Additionally, clinicians were found to be protective of the autonomy and privacy of patients, and fearful of the legal consequences for themselves. Turner et al^64^ evoke an inclusivity argument, drawing upon restorative just culture (RJC) to ‘focus upon the hurts, needs and obligations of all who are affected by the event’ (a summary of RJC in response to incidents is below in Box 5). Bouwman et al's^74^ work regarding carer involvement suggests there are ‘no easy answers and solutions available’ and careful consideration is needed.

Box 5 Summary of response to incidents, using a restorative just culture framework (adapted from Turner et al^64^)

**Table:** 

Who is hurt? Consumer, family, carer, clinician and organisation.
What do they need? Tailored to each individual, consideration may encompass: support, healing, information, engagement in review and learning.
Obligations and actions For all affected there are obligations and actions required by the organisation to provide transparency about what has happened, inclusive involvement in review processes, necessary high-quality learning is made and appropriate emotional support is provided throughout.

#### Competing hierarchies of knowledge: technical–rational and experiential

Debate is evident in the literature as to whether investigation processes are driven by central government mandates and therefore seen as good governance by local managers, whether they are explicit measures to promote patient safety or both.^54^ From a psychoanalytic perspective, Cohen's^63^ editorial piece makes a cogent argument for the (RCA) investigative process to represent a systematic, objective and measurable response to serious incident investigations. In doing so, the organisation is situated as a legitimate power in the management of ‘reputational risk’.^63^

Matters that involve the personal, subjective and emotional are deprivileged and represent potential threats to the dominant (rational) orthodoxy. Fröding et al^70^ provide contemporary evidence of this top-down approach.

In contrast to the dominance of technical–rational approaches to serious incident investigations, Turner et al^64^ take RJC as a rallying point for reimagining the workplace culture, running in parallel with a ZSF. On the one hand, their paper highlights the need to safeguard the welfare of clinicians as second victims. On the other hand, the importance of the clinician's experience is held up as a potential source of organisational learning, commensurate with a corresponding shift from Safety-I to Safety-II.^64^

#### Development of the patient safety agenda

The literature includes suggested improvements to existing approaches (Canham et al,^67^ Fröding et al,^70^ Gillies et al,^66^ Jun et al,^73^ Turner et al,^64^ and Wyder et al^46^). Clarke^54^ points out that despite some reservations about RCA, it has at least heralded a move toward systems analysis rather than targeting individual practice; a corresponding shift in vocabulary with ‘critical incident review’ becoming subsumed under a broader heading of ‘patient safety’. The literature is beginning to show that more authors from outside healthcare, but with expertise in safety science, are writing about patient safety in mental healthcare (Canham et al,^67^ Jun et al^73^ and Turner et al^64^).

Wyder et al^46^ argue for the utility of triage tools to systematically assess the most helpful way of investigating. Additionally, Gillies et al^66^ developed a taxonomy of suicide-related factors for patients who had died within 7 days of contact with services. They propose that this would help services standardise their reviews and in turn advance widespread improvements. Wyder et al^46^ also advocate aggregating investigation findings as an aid to organisational learning. Aggregation of data remains indicative within Turner et al's^64^ perspective, as does triage.

A recognition has emerged that Safety-II can be a successor to Safety-I, emphasising the value of establishing a critical understanding of ‘what went right’, but also shining a spotlight upon human variance and clinical innovation in the face of complexity and uncertainty.^75,76^ Jun et al^73^ illustrate this by bringing systems theoretic accident modelling and processes (STAMP) analysis^77^ to a Safety-I approach, and clinician interviews to a Safety-II approach. The STAMP model, also advocated by Canham et al,^67^ reworked suicide prevention processes as safety control structures through the analysis of 41 RCA reports. Specific attention is given to control feedback loops within an organisational structure that do or do not respond to service delivery standards and the status of the patient. Their results advocate both approaches as assets to patient safety. Fröding et al^70^ also endorse benefits of a Safety-II perspective, although it is less clear if this is parallel to or a replacement for a Safety-I approach.

As part of a move away from the Safety-I retrospective analysis of ‘what went wrong’, Turner et al^64^ twin-track the development of a RJC alongside a ZSF as a more inclusive way to foster a systems approach. To overcome the gap between work as imagined and work as done, Turner et al^64^ evoke the notion of double-loop learning^78^ as a mechanism through which the two can be more closely aligned. However, this is not the first mention of double-loop learning and a focus on culture in this literature. Clarke^54^ having previously cautioned that such an approach is likely to rest on both organisational and clinician maturity; implicitly and explicitly recognising the primacy of learning and being open to criticism. Turner et al's^64^ restorative approach also provides a tangible response to earlier concerns raised by Cohen^63^ regarding the importance of ‘emotionally involved practice’.

### Discussion

In summary, there is a dearth of empirical research specific to the quality of serious incident investigation following suicide within adult community mental healthcare; a matter that Wyder et al's^46^ review describe as a ‘real concern’. Of the eight empirical papers included in this study, only Canham et al^67^ and Jun et al^73^ specifically explore community-based suicide. Bouwman et al,^74^ Fröding et al^70^ and Vrklevski et al^65^ claim their respective papers as ‘firsts’ in the field. Indeed, several papers are described as exploratory,^70,72,74^ which, given their relative recency, attests to the immaturity of the knowledge base.

Much of the empirical literature reported here adopted a qualitative approach and drew upon a range of appropriate methods (e.g. documentary analysis, semi-structured, one-on-one interviews). A range of analytical tools were also evident, which often reflect the researcher's scholarly background. Our discussion synthesises the findings in line with the model chosen^43^ and each of the review objectives. Under each objective, we will consider the who, how and why. Our objectives and related responses should not be seen as mutually exclusive.

#### The nature and extent of relevant individuals’ involvement in investigations

Bouwman et al^74^ evidence the value of carer involvement in the healthcare and risk assessment process but significant challenges have been reported.^74,79^ However, the extent and level of carer involvement in serious incident investigations, whether positive or negative, is unknown.

Healthcare policy has again driven this aspect of healthcare governance in the UK, starting with ‘Being Open’,^80^ and the recent introduction of PSIRF within England,^32^ offering guidance for those affected, including carers. However, the recommendations regarding engagement with carers incorporated within PSIRF are not mindful of complex mental health incidents such as suicide, and require further exploration and research^79^ concerning the epistemological and methodological basis underpinning carer involvement.

How clinicians should be involved in investigative processes has long been contested. There is a need to engage clinicians in a holistic way to maximise organisational learning while safeguarding their personal and professional well-being. It appears there is no easy solution as any investigation must generate some analysis of professional practice.

Although a contemporary systems approach to investigative work seeks to accommodate the idea of work as done in counterbalance to work as imagined, it is predominantly imagined by regulators, coroners, managers or indeed carers. A more conducive environment to openly support clinicians would seem to be a minimal requirement for meaningful learning as indicated in Sandford et al's^81^ systematic review.

More broadly, the question of which professionals should be involved and whether this should span across agencies is only minimally discussed within the reviewed literature, despite the inevitable involvement of numerous parties. Fröding et al^38^ recommend the inclusion of multidisciplinary analysis teams across organisational boundaries.

There is a wholesale absence of investigators’ personal perspectives on the serious incident investigation process in the reviewed literature. This is concerning given that they are identified as ‘third victims’^12^ and are likely to have valuable insights. It is therefore prudent for organisations to consider the role of restorative clinical supervision.^82^ Given the multiple tensions within the investigative process, gaining an understanding of how investigators grapple with them is an essential area for exploration. Cohen^63^ hypothesised that investigators discharge their role with a need to protect the agency and/or themselves. Fröding et al^38^ suggest the need for education and training for investigators within a wider range of suggestions for improving learning from suicide investigations.

We have seen how the role that the organisation takes in this process is unlikely to be value-free, often defaulting to a top-down process that could negatively affect the learning generated. Contemporary mental health organisations are also influenced from national policy directives (e.g. SIF and PSIRF), supporting the point that broader hierarchical influences affect the organisation.^67^

#### Appraise the strengths, limitations and evidence base underpinning the approaches taken

Nationally and internationally, many of the concerns about investigation approaches remain unanswered by empirical examination or service evaluation; illustrated by the RCA method, despite widespread utilisation and historical concerns.^55^

Vrklevski et al^65^ question whether organisations are using the RCA model correctly, as there may be variation in how it is applied. Consequently, this limits the strength of conclusions made from the selected literature, which seek to amalgamate findings from numerous RCA reports.^46,72^ We argue that some RCA critiques are not pitched at the approach *per se*, but more aligned with administrative and peripheral factors;^83^ an argument that can be applied to the critique of early approaches.^56^

Dekker^84^ defends RCA in disentangling complexity, although with limitations. Snowden^85^ also acknowledges limitations of RCA, although states that there will be some cause-and-effect pathways to which RCA is sensitive. Importantly, he also proposes several options for improving RCA, including consideration of cognitive biases, recognition of constraints and conflicts in finding causes, analysis of investigators’ knowledge application and mapping staff attitudes to the investigative process.^85^

There is a challenge in bringing together a range of discursive positions around theory and practice. Within the patient safety domain, the lack of shared meaning represents a barrier to progress.^86^ The work of Canham et al^67^ and Jun et al^73^ represent important progressions where clinicians and safety modelling experts collaborate. Yet, the absence of suicide risk experts in their work and their appreciation of work as done is noteworthy. In contrast, Turner et al^64^ provide a multidisciplinary authorship that offers promise of achieving a truly tailored approach to this vital aspect of mental healthcare.

Although some of the literature noted limitations with suicide risk assessment,^55,72,73^ concerningly there was little examination of underpinning concepts and the evidence base. Turner et al^64^ argue that investigatory approaches are often undertaken ‘through the lens of risk prediction, implying that an improved risk assessment could have led to a different outcome’. Indeed, in the UK, the NCISH^4^ and National Institute for Health and Care Excellence^87^ recommend against the use of risk assessment tools to predict suicide.

Contemporary approaches to assessing suicide risk highlight the importance of collaboration with patients and carers, building a therapeutic relationship and using this to inform preventative interventions.^4,67,87–93^ However, this work appears absent from any proposed investigatory evidence base and subsequent practice guidelines. Although these approaches appear recent, they each have origins in an established literature. The inertia in incorporating these approaches within investigative methods is concerning. This is alluded to by Hawton et al,^88^ who comment upon a reluctance to move away from risk prediction in mental health services. Their conclusions echo Cohen,^63^ who suggested prediction may have a protective function against organisational anxieties a decade earlier.

Turner et al^64^ were the only authors to describe utilising a preventative formulation-driven approach^92^ within their system-wide framework that prioritises learning. Positive findings in relation to the recommendations generated have recently been published.^94^ Given the significant lack of attention in this area, we argue the need for further research.

#### Consider the influence of various investigative approaches upon organisational learning

Following the popular maxim that the answer is only as good as the question, any learning generated to inform organisational improvement will only be as good as the breadth and depth of the investigatory process.

The well-reported complexity of services and the equally complex risk judgements provide a strong argument for more sophisticated, yet systematic methods of learning. Given the preoccupation with traditional investigative methods, some fundamental opportunities for organisational learning are lost.

The limitations of RCA inevitably have an impact on the learning generated. Braithwaite et al^35^ highlight that it is unable to grapple with the complexity and unpredictability of healthcare. Further, the identified gaps in evidence-based approaches to the involvement of all relevant individuals clearly have significant potential for creating gaps in learning. The inappropriateness of RCA in the investigation of suicide is further highlighted by Fröding et al,^38^ who note the key, but unknown agency of the patient. In line with our arguments, Fröding et al^38^ also suggest that contemporary models of suicidal behaviour and preventative approaches should be utilised.

Averill et al^39^ draw attention to the need to understand the patient journey and the potential for iatrogenic harm, which may inform future investigation processes. In relation to a proximal focus of suicide investigations, Reason^95^ asserts that incidents often ‘have a causal history that extends back in time and up through the different levels of the system’. Similarly, Turner et al^94^ took a ‘learning anything’ approach to their incident response framework.

Cohen^63^ and Turner et al^64^ raise the quality of the therapeutic relationship and its absence from the investigative process because it is perceived as unreliable, especially within a RCA framework. This is concerning given that the quality of the therapeutic relationship is highlighted as being associated with suicidality,^96^ and those at high risk of suicide are likely to experience significant difficulties in engaging with clinicians.^97^ It is reported that suicidal patients may avoid any disclosure because they do not want to be a burden, experiencing shame or stigma,^98^ therefore placing fundamental importance on the therapeutic alliance.^99^

Furthermore, Safety-I methods may need to be complemented by those of Safety-II. In recognition of the limitations of RCA, Braithwaite et al^100^ flag the importance of future research appreciating what helps things to go right (Safety-II). Although the authors do not reference the implementation of Safety-II, practical examples are now appearing in the literature.^101^ The need to shift to systems-based models of approaching healthcare investigations and the potential benefits for learning are discussed by Sampson et al^37^ and Weaver et al.^102^ Their work references the Systems Engineering Initiative for Patient Safety framework^103^ as proposed in the UK's PSIRF.^36^ However, the application of this model to the complexities of suicide under adult community mental health services requires evaluation. More broadly, this review has highlighted the importance of Safety-II approaches being underpinned by a system culture that incorporates ZSF and RJC. Indeed, this approach has demonstrated some early positive findings in suicide prevention outcomes.^93,94,104^ The PSIRF is grounded in the concept of a ‘just culture’,^105^ and although this has some elements of RJC, it does not mandate that learning and improvement should prioritise the healing of all involved. Turner et al^64^ document their concerns about a just culture approach.

We argue that a weakness in these approaches concern the possible tensions for professionals should regulatory bodies and employing organisations hold opposing views. Therefore, it will be important for future work to also consider the impact of broader policy and regulatory processes on healthcare services more widely.

It is also pertinent to acknowledge concern about the implementation of zero suicide approaches at a service level. Mokkenstorm et al^106^ discuss the potential for inducing guilt in clinicians and carers, which could have an adverse impact on the openness of those who have been close to the deceased and, in turn, the completeness of learning. To resolve this, the authors conclude that a ZSF must be located in a RJC.^64^ Turner et al^94^ provide evidence to mitigate any concerns about the ZSF with their early findings that a supportive culture can be a protective factor for staff. The need for evaluation of carer experience is identified as a priority for future research by the authors.

Additionally, any investigation must be able to grapple effectively with wider contextual factors. Within England, the demand for mental health services are outstripping resources and the workforce needed to provide services,^107–109^ an issue further compounded by a broader health and social care system that is described by the national regulator as ‘gridlocked’,^110^ where ‘good safe care’ has been compromised by underinvestment. This poses a challenge for the mental health workforces internationally, already experiencing reportedly high levels of burnout.^111^ This is concerning given Canham et al^67^ and Jun et al's^73^ findings that clinicians draw upon personal resources when undertaking suicide risk decisions and developing deeper relationships with patients.

Various literatures contend that the RCA process is resource intensive,^112,113^ but alternatives will also challenge organisational resources especially those requiring cultural change. Surprisingly, the reviewed literature did not explore this theme. However, the necessity of resource management makes the case for deeper investigations of lower numbers of incidents rather than superficially investigating many.^114^ This argument further enhances the case for an associated triage process.^38^

Concerning triage, and to improve the quality of learnings generated from suicide investigations, Fröding et al^38^ propose the need for improvements in the involvement of patients and carers, education and training for investigators, and multidisciplinary analysis teams working across organisational boundaries. In relation to Fröding et al's^38^ recommendation of multiagency team involvement, we found a lack of discussion within the reviewed literature regarding who to involve in the process and how.

Drawing upon Iedema et al's^115^ empirical work, Cohen^63^ draws attention to the role of the investigator, and the potential constraints within the investigatory process itself that promotes aspects that are ‘practical, sensible and achievable’, as well as demoting others that are seen as ‘ambiguities, incommensurabilities and conflicting goals’. Such contradictions and their ethical consequences demand qualitative research to generate a deep understanding about how investigators undertake their role. We contend that to do so requires delicate balance to be achieved between governance and reputational management, and it is this that is centre stage, not necessarily the dominance of traditional investigative methods.

Nicolini et al^116^ suggest that a shift away from governance and legitimation is necessary to generate high-quality organisational learning. Their approach chimes with the work of Turner et al^64^ and corresponds with the UK's PSIRF.

### Quality assessment of the robustness of the synthesis

Principal limitations of integrative reviews and syntheses include a lack of transparency, particularly in terms of method and reducing selection bias; the quality of studies selected; and the potential subjectivity of content analysis and theme generation.^117^

We address transparency through a detailed description of our search strategy: co-developed and overseen by an NHS information specialist and two carers, the setting of exclusion criteria, and an iterative review and selection process, subjected to multidisciplinary peer review (H.H., T.S., G.A.).

**Fig. 1:**
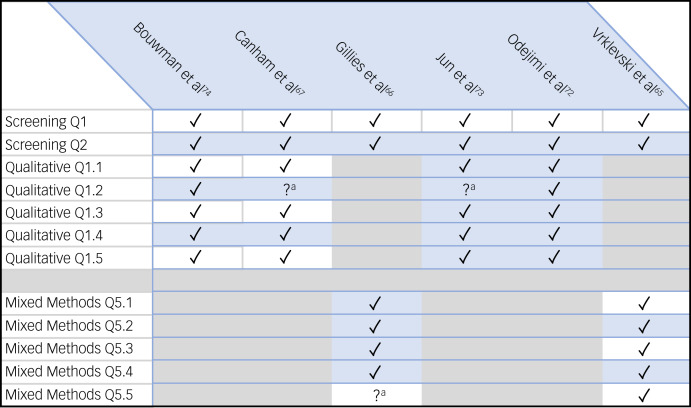
Mixed Methods Appraisal Tool appraisal (Hong et al^51^). ^a^Appraisal was limited due to this aspect of the study being less detailed.

To address the quality of empirical studies selected, the MMAT^51^ was applied to six of the eight empirical papers reviewed in this study. As literature reviews, the Wyder et al^46^ and Fröding et al^70^ papers are outside of the remit of the MMAT. The majority of papers fully complied across all domains of the MMAT, but some were less detailed, which limited our appraisal (see Fig. 1).

The non-empirical work here is authored by a range of senior clinical and/or research experts in the specific field, and their published papers draw upon a range of empirical literature to support their perspectives. We argue that the selected papers demonstrate a sufficient level of reflection, critical analysis and evaluation to warrant inclusion.

Subjectivity was addressed through an iterative procedure, periodic consultation with carer representatives, who reviewed the penultimate draft, as did an independent NHS suicide prevention lead and serious incident investigation lead. After Gove et al,^118^ carer involvement with the analysis and synthesis processes may have further qualified our findings. Finally, our review was informed by the Scale for the Assessment of Narrative Review Articles (SANRA) framework^119^ (see Supplementary Appendix 2).

In conclusion, suicide is an ultimate harm that embodies the unpredictability and complexity of human behaviour; establishing causation is neither simple nor certain. The contemporary international research and additional grey literature reported here provides evidence into how investigatory approaches based on Safety-II approaches can be aligned with RJC and ZSF. These approaches have contributed to better outcomes in terms of patient safety.

This review casts the dominant RCA approach as a largely inappropriate investigative tool, and questions remain about its suitability for suicide investigations as part of a patient safety paradigm. However, the limitations of RCA are one part of an underdeveloped, largely unevaluated approach to the investigative process and the way in which healthcare providers can learn from suicides.

Embracing suicide prevention as a fundamental outcome, we argue that future research must attend to a greater understanding of all people affected by suicides that occur in adult community mental healthcare. This includes serious incident investigators and their managers, clinicians, carers and the patient-in-context. Moreover, research needs to be sensitive to the enduring determinants of suicide, particularly at a time where the longer-term consequences of COVID-19 are uncertain. Finally, attention must also be directed upstream, at the broader influences affecting healthcare organisations and regulatory bodies, and how these may shape opportunities for improving the patient safety agenda in this domain.

## About the authors

**Helen Haylor** is a Service Evaluation Lead with the First Response Crisis Service, Bradford District Care NHS Foundation Trust, UK. **Tony Sparkes** is an Assistant Professor at the Faculty of Management, Law and Social Sciences, University of Bradford, UK. **Gerry Armitage** is a Research Advisor at the Research and Development Department, Bradford District Care NHS Foundation Trust, UK; and Emeritus Professor at the Faculty of Health Studies, University of Bradford, UK. **Melanie Dawson-Jones** is a Knowledge Manager with the Library and Health Promotion Resources Centre, Bradford District Care NHS Foundation Trust, UK. **Keith Double** is an Involvement Partner with the Patient and Carer Experience and Involvement Team, Bradford District Care NHS Foundation Trust, UK. **Lisa Edwards** is a Carer Representative and an Assistant Professor with the Faculty of Health Studies, University of Bradford, UK.

## Supporting information

Haylor et al. supplementary material 1 Haylor et al. supplementary material

Haylor et al. supplementary material 2 Haylor et al. supplementary material

## Data Availability

Data availability is not applicable to this review article.
