# The process and perspective of serious incident investigations in adult community mental health services: integrative review and synthesis – ERRATUM

**DOI:** 10.1192/bjb.2024.1

**Published:** 2025-02

**Authors:** Helen Haylor, Tony Sparkes, Gerry Armitage, Melanie Dawson-Jones, Keith Double, Lisa Edwards

**Keywords:** Suicide, community, serious incident investigation, organisational learning, patient context, erratum

This article was published with a number of errors which occurred during the proofing process, which the publishers apologise for. These have since been corrected within the article and the changes made are listed below.

Table 2 was originally published without the flowchart element, this has now been updated.

**Table 2:** 

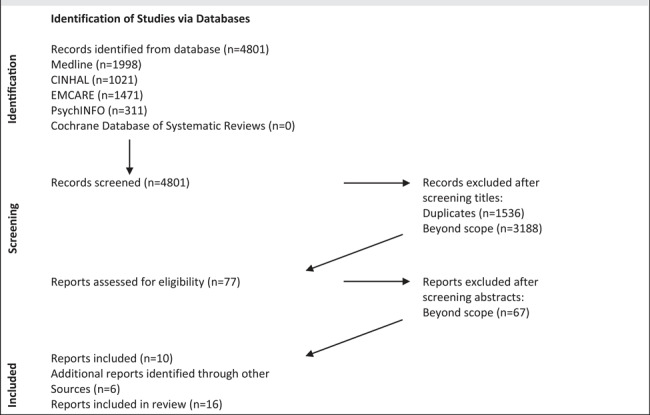

Boxes 1, 2 and 5 were unclear in their presentation. The correct presentation is below:

Box 1: Lead: **System change occurs with sustained and committed leaders who learn and improve practices following adverse events.**

Train: **Train all staff—clinical and non-clinical—to identify individuals at risk and respond effectively, commensurate with their roles.**

Identify: **Screen and assess every new and existing patient for suicidal thoughts and behaviours in an ongoing and systematic way using standardized tools.**

Engage: **Patients at risk for suicide agree to actively engage in a package of evidence-based practices that directly targets their suicidal thoughts and behaviours.**

Treat: **Utilize evidence-based treatments that focus explicitly on reducing suicide risk to keep patients safe and help them thrive.**

Transition: **Put policies into action that ensure safe hand-offs between caregivers and upon discharge.**

Improve: **Apply data-driven quality improvement. Use data to inform system changes that will lead to improved patient outcomes and better care for those at risk.**

Box 2: • Learning from practices that fall outside of internal service review/audit activities.

• Specific service user populations and/or specific clinical conditions.

• Specific professional groups.

• Self-harm.

• Hospital (non-community) based mental healthcare.

• Service-level cultural context of SIIs and how they are reported.

• Outcomes, recommendations, and implementation of recommendations in relation to suicide investigations.

Box 5: Who is hurt?

Consumer, family, carer, clinician and organisation

What do they need?

Tailored to each individual, consideration may encompass: Support, healing, information, engagement in review and learning.

Obligations and actions

For all affected there are obligations and actions required by the organisation to provide transparency about what has happened, inclusive involvement in review processes, necessary high-quality learning is made and appropriate emotional support is provided throughout.

The numbering of some of the references was also incorrect. The citations which are corrected are 51–57 and 72.

Terms such as “Safety-I” and “Safety-II” were inconsistently capitalised and have been corrected.

Appendix I was missing from the supplementary material. This has now been added and is available at https://doi.org/10.1192/bjb.2023.98.

A dedication has been added to the Acknowledgements. In memory of David Edwards-Gill and Stephen Sanderson, loved ones lost to suicide, who continue in their legacy to inspire this work.
