# Supplementary material for: The process and perspective of serious incident investigations in adult community mental health services: integrative review and synthesis
Source: BJPsych Bull. 2025 Feb;49(1):23–35. doi: 10.1192/bjb.2023.98 (PMC11810474; doi:10.1192/bjb.2023.98)
Supplement: Haylor et al. supplementary material 1 — Haylor et al. supplementary material [file S2056469423000980sup001.docx]

**Appendix I**

**Summary of empirical studies with a focus upon serious incident investigations (n=8)**

| **Author(s), year and country** | **Aims** | **Design/methods/Sample** | **Findings** | **Conclusions** | **Limitations/notes** |
| --- | --- | --- | --- | --- | --- |
| Bouwman et al^74^  Netherlands | To explore the role of patient and family involvement in formal processes following suicide. | Qualitative approach involving multiple methods; incorporating documentary analysis and 1:1 stakeholder interviews (n=35). | Patient and family involvement have little representation in policy documents. In practice involvement consists of aftercare provision and post-event information sharing.  Privacy and involuntary admissions are held as a barrier to involvement.  Involvement should not be mandatory and involvement during the whole treatment process would be helpful, with early involvement especially so. | In terms of policy, involvement linked more to routine care than sentinel events.  In practice, involvement is limited to aftercare and information provision, with analysis an internal, organisational, matter.  More needs to be done. | Potential for response bias and recall bias.  Difficulties with recruitment and no controls over the context of the event.  An explorative piece of work, and one of the first in this domain. |
| Canham et al^67^ UK | As an alternative to Root Cause Analysis (RCA), to (re)analyse suicide incident reports using Systems Theoretic Accident Modelling and Processes (STAMP) as a way of generating a deeper understanding of suicide incidents.  RCA conceptualises incidents as linear, rather than a complex set of issues emerging from relationships within the system. | A qualitative approach.  Investigations involving RCA were (re)analysed (n=41) using STAMP. | Weakness within the system safety control structure:   - Control-feedback loop undermined by reduced ability to understand and predict dynamic risk in the absence of continuous observation. - Loss of clinical feedback due to engagement issues and adherence to treatment plans. - Meaningfulness and subsequent take-up of treatments. - Knowledge of new patients is often incomplete. - Communication and coordination issues within the care process.   Area 5 is played out over longer periods of time | Community mental health care is a challenging environment to monitor and control suicide risk.  Such challenges fit with an ultra-adaptive safety model.  The ability and capacity of community mental healthcare to adapt to patient need is questioned.  Adaptive capacity may be realised thorough the creation of buffers, but this may not be consistent with a lean philosophy.  Patient engagement and service design in need of further research | Access to an ergonomics researcher.  Explicit focus upon community cases. |
| Fröding et al^70^ Sweden | How mandatory reporting of suicides has influenced:   - Associated investigations. - Lessons learned. - Improvements to patient safety. | Retrospective study of SII reports (3x cohorts: 3x points in time)  279  436  316  n=1031  (13 years) | Investigations took a microsystem perspective, looking predominantly at final contact.  Recommendations included:   1. Updating existing or developing new routines. 2. Educational actions.   But recommendations did not include:   1. Sharing information across departments. | Investigations:  Restricted to passing information to the supervisory authority (rather than safety focussed).  Investigations suited the structure of the reports rather than the incident.  No new service improvements or lessons identified! | One of the first papers to evaluate outcomes of investigations of harm (in this case suicide).  The quality of the investigative analysis not evaluated.  Only one researcher collected and categorised data, hence open to bias. |
| Gillies et al^66^ Australia | (a) develop a standardized taxonomy for  RCAs;  (b) to quantitate service-related factors associated with suicides; and  (c) to identify service-related suicide prevention strategies | Qualitative approach involving the thematic analysis of Root Cause Analyses (n=64).  People who died within one week of contact with services over 5 yrs (2008-12) | Super and sub-ordinate themes:   1. Individual factors    1. Reason for referral       1. Suicidal ideation       2. Depression       3. Suicide attempt       4. Psychosis       5. Self harm    2. History       1. Suicidal ideation       2. Depression       3. Suicide attempt       4. Psychosis       5. Self harm       6. Drug and alcohol misuse 2. Situational factors    1. Stressors       1. Relationships       2. Health       3. Loneliness or loss       4. Financial stressors    2. Support       1. Client responsible for attending support services       2. Being cared for by family or friends       3. Identified support needs not addressed 3. Care-related factors    1. Risk assessment       1. Denial of suicidality       2. Communication with clinician       3. Communication with other services    2. Medication 4. Changes in medication 5. Poor adherence 6. Side effects 7. Follow-up 8. Lack of follow-through 9. Unintended appointments 10. Phone contact only   The most common factor was that clients had recently denied suicidality.  Reliance on carers, recent changes in medication, communication problems, and problems in follow-through were also commonly identified. | The most common factor was that clients had recently denied suicidality.  Given the difficulty in predicting suicide in people whose expressions of suicidal ideation change so rapidly, services may consider the use of strategies aimed at improving the individual, stressor, support, and care factors identified in this study. | Due to complexity, assessment needs to be multidimensional and involve all informants.  The role of carers is important.  Suicide within 7 days of contact.  No case controls. |
| Jun et al^73^ UK | Comparing safety I & II approaches to detection and response process for suicide prevention. | SI = 41 SIIs (based upon RCA) analysed using a systematic analysis approach using Systemic Theoretic Accident Modelling and Process (STAMP).  SII = 20 interview with community MH practitioners and managers. | SI =   1. Inherent weakness in the patient/clinician interactions and uncertainty about risk detection. 2. Poor engagement with services. 3. Reliance on patients self-presenting in crisis. 4. Delay in treating new patients. 5. Co-ordination, communication and process issues   SII = Complex decision-making process in context of uncertainty and fluctuating risk. Three areas for consideration:   1. Being patient centred. 2. Individual and service level consideration of resource availability. 3. Legal and procedural responsibility. | SII indicates valuable insights to improve the system without compromising it.  SI indicates systemic issues and raises questions about dealing with them.  Both SI and SII therefore important.  SII findings are in line with Vincent and Amalberti^*^  3 main approaches:  Ultra-adaptive  High reliability  Ultra-safe  The safety approach in community MH care is likened to ultra-adaptive model.  To improve safety in a certain type of system – Ultra Adaptive system - Embracing Risk   - Power to experts. - Training through peer-to-peer learning. - Shadowing. - Acquiring professional experience. - Knowing one’s own limitations. | Contribution of Safety I and II approaches to process.  Safety I method/analysis draws upon Canham et al^67^ |
| Odejimi et al^72^ UK | To explore common themes from Root Cause Analyses of Serious Incident reports. | Qualitative case review (n=48) and thematic analysis. | 3 superordinate (and interwoven) themes:   1. Patient related factors 2. Exacerbated mental health conditions. 3. Lack of engagement with services. 4. Non-adherence to medications. 5. Professional 6. Risk assessment and management. 7. Inadequate clinical enquiries. 8. Non-adherence or poor adherence to policies and procedures. 9. No interprofessional communication and collaboration 10. Lack of consultation of carers by clinicians. 11. Organisation 12. Inadequate psychiatric accommodation. 13. A lack of additional support for mental health patients (such as drug and alcohol services). 14. Unsafe wards.   Only 1 death in the hospital setting | Majority of the  deaths were caused by patient-related factors, particularly exacerbation of the patient’s mental health condition, and patient assessment and management.  RCA in their analysis is an improvement over the SAD PERSONS scale. As it includes professional and organisational factors. | Supportive of RCA.  Carers involvement is important, along with a person-centred approach. |
| Vrklevski et al^65^ Australia | Evaluate the impact of RCA upon patient outcomes, and to ascertain whether it is I the most appropriate model.  How many recommendations from RCAs are implemented and  in what time frame?  2. What are the challenges and issues in implementing recommendations  across the mental health service?  3. What are the barriers in uptake and implementation of recommendations  from RCAs?  4. What types of recommendations are more likely to be implemented  and what types of recommendations are less likely to be implemented and why? | Mixed-method design (both qualitative and quantitative methods).  Review of RCA literature.  Quantitative data extracted from RCA database and subjected to frequency analyses.  15 semi structured interviews with managers (n=5), RCA team leaders (n=5) and clinicians (n=5); transcripts subjected to discourse analysis. | 70% of RCAs did not lend themselves to establishing a root cause.  Low clinical engagement in recommendations.  Poor evidence that the implementation of recommendations leads to safer patient care.  Troubling: recommendations are repeated (that reflect existing policy or previous recommendations). | Although the RCA model offers a formal and systematic approach to the review of serious critical incidents in mental health, it is not the model of best fit.  Only 65% of recommendations made through RCA reviews are implemented within 12 months. | The first formal review of RCA in NSW Australia. |
| Wyder et al^46^ Australia | Synthesis of literature re: findings of system errors in context of suicide deaths. | A systematic narrative meta-synthesis.  PRISMA methodology. | 14 papers (9 inpatient/5 community deaths).  Vulnerabilities in the patient journey:   1. Inappropriate/incomplete risk assessments. 2. Lack of family involvement. 3. Transitions and communication between teams. 4. Policies and procedures not always followed. 5. Treatment not in line with current guidelines. 6. Access to means and observation. 7. Lack of specialist services within the community.   Other than:   1. Enhancing policy. 2. Guidelines. 3. Documentation. 4. Regular training.   Limited suggestions for system improvement. | Limited studies investigating learning and recommendations. | Inductive. |

^*^Vincent C, Amalberti R. *Safer healthcare: strategies for the real world*. 2016. Springer.

**Summary of non-empirical studies that focus upon serious incident investigations (n=8)**

| **Author, year and date** | **Format and aims/purpose** | **Arguments and main points raised** | **Conclusion** | **Comments** |
| --- | --- | --- | --- | --- |
| Amos and Shaw^52^ UK | Editorial on the broad issue of reviewing serious incidents at a time of the National Service Framework for Mental Health. | Local reviews c/w national reviews.  Benefits of local reviews:  Detailed peer review (including out of the health field).  Local relevance.  Comparison with national data.  Limitations:  Small numbers.  Local services may not be aware (of some suicides). | Learning from experience a priority. | An early paper that harnesses the NSFMH to raise the utility of ‘local’ reviews of SIIs. |
| Catalan et al^53^ UK | Commentary.  Clinical audit (systematic).  Description of a multi-disciplinary suicide audit process. | Although couched as commentary, the paper offers a systematic approach to the implementation of a clinical multidisciplinary audit of suicide in a general psychiatric service’ (p184) Results are discussed in terms of:   - Characteristics. - Discussion of circumstances surrounding each suicide and recommendations. - Assessment and management of suicide risk within the mental health service. - Access to means. - Aftermath. | A feasible process providing a:  Place and space to review the case.  Opportunity to reflect on the difficulties in suicide prevention.  Links made to NCISH (1999).  Local impact. | The process described is not linked to managerial inquiries.  Similarities to Rose^57^  Problem focus?  Systematic focus; culture of blame. |
| Clarke^54^ UK | A discussion paper on the utility of the ‘critical incident review’. | Historical basis to Incident investigation: the ideal, and the ‘learning organisation’ and Root Cause Analysis discussed.  Historically: failure of community care and critique of mental health services > sensible approach > a scientific approach (via RCA and ‘think tanks’.  Patient safety as a metaphor for critical incident review. | Definite solutions to adverse events are an illusion, but learning through mistakes requires serious and systematic study. | Good historical account.  The management of risk needs to be good practice and part of the culture not something outside of this. Everybody’s business.  SIIs are unhelpful?  An ideal method is a scientific method. Root cause analysis?  Double loop learning? |
| Cohen^63^ UK | Editorial | Structures and processes can fit with organisational defences regarding suicide. Actor-network theory taken to better understand this. | Organisational defensive processes.  Demonstrable legitimacy and the ‘risk management of everything’.  Blame and guilt and the ‘omnipotent fantasy’ of prevention – an organisational defence.  Actor network theory to explain causes and consequences at an individual level. | Some good comments and well-referenced.  Depersonalised language – in keeping with a mentality that is legitimate and approved. |
| Neal et al^55^  UK | Opinion.  Root cause analysis as a means to an end. | RCA as an efficient and consistent means of analysis? But as yet untested. | Useful for understanding RCA in a practical sense. | Dated?  1^st^ level  2^nd^ level  Root causes  Limitations (critique of RCA)  Benefits  10 step process  Link to Rose^57^ |
| Rose^56^ UK | Opinion  Commentary on the 6 year ‘Oxford’ project (1994-99). The project aimed to offer:  A systematic approach to learning lessons and applying the learning to practice.  To identify patterns over time.  Conversation about how to provide the best care in the safest way. | Structure of the review is described; a summary of all incidents reviewed over the period, and the main learning points.  Strengths and limitation are noted. | Main lessons:  Reducing means of self-injury.  Improvement of care quality.  Training.  Clinical practice and procedures.  Staff needs.  Strengths:  Systematic approach.  Micro analysis in collaboration.  Care quality debates (appreciative).  Difficulties:  Individual case review needs to have been undertaken first.  Maintaining the inertia.  Perception of the review from carers and relatives. | The Oxford project had a particular focus upon psychiatric inpatients; not wholly specific to suicide, but an early commentary on the dearth of literature about how to investigate the deaths of patients.  A detailed, progressive multi-agency, interprofessional collaborative review process.  Seek to identify common themes.  Local findings acknowledge national inquiries.  Reference made to  Appleby et al^62^  NB typo? N=86 should read n=63  But, @>50% may be attributable to suicide.  How does the review sit in regards to: formal review, individual case review? |
| Rose^57^ UK | Review of the Oxford SIR.  Conceptual. |  | As above. | Not all bad clinical outcomes can be put down to system problems. Individuals can make serious mistakes or be negligent and need to be held accountable. |
| Turner et al^64^  Australia | Review.  To demonstrate that a focus upon Safety II through a Zero Suicide Framework and Restorative Just Culture are best placed to provide the needed shift away from Safety I. | Challenges:   1. To the fallacy of risk prediction. 2. To incident reviews that maintain a retrospective linear focus (and influenced by hindsight and outcome bias).   7 essential elements of ZSF  Leadership  Train  Identify  Engage  Treat  Transition  Improve  Current arrangements ignore the complexity of the healthcare setting.  These issues have to be identified, reconciled and integrated into future pathways.  A Zero Suicide Framework can help to transition from the retrospective focus on errors (Safety I), to a more prospective focus upon complexity (Safety II) if wedded to a Restorative Just Culture (that has a focus upon hurts, needs and obligations of all who are affected by the event.  Workplace cultural change is imperative. | Main lessons  RJC as a foundation to ZSF can manage the anxiety and blame culture associated with critical incidents.  RJC allows for effective involvement of all. Overcomes risk prediction and other retrospective biases.  Leaders at national, state and local level need to take accountability for required cultural changes required for staff and patient safety, | First paper which brings together experts in approaches to suicide risk assessment, clinical experts and patient safety  Provides response to Cohen’s paper flagging defence against anxieties. |
