# Supplementary material for: The process and perspective of serious incident investigations in adult community mental health services: integrative review and synthesis
Source: BJPsych Bull. 2025 Feb;49(1):23–35. doi: 10.1192/bjb.2023.98 (PMC11810474; doi:10.1192/bjb.2023.98)
Supplement: Haylor et al. supplementary material 2 — Haylor et al. supplementary material [file S2056469423000980sup002.docx]

**Appendix II**

2

2

2

2

2

2

12
